# Deep Sequencing of HIV-1 near Full-Length Proviral Genomes Identifies High Rates of BF1 Recombinants Including Two Novel Circulating Recombinant Forms (CRF) 70_BF1 and a Disseminating 71_BF1 among Blood Donors in Pernambuco, Brazil

**DOI:** 10.1371/journal.pone.0112674

**Published:** 2014-11-17

**Authors:** Rodrigo Pessôa, Jaqueline Tomoko Watanabe, Paula Calabria, Alvina Clara Felix, Paula Loureiro, Ester C. Sabino, Michael P. Busch, Sabri S. Sanabani, for the International Component of the NHLBI Recipient Epidemiology and Donor Evaluation Study-III (REDS-III)

**Affiliations:** 1 Virology Department, São Paulo Institute of Tropical Medicine, University of São Paulo, São Paulo, Brazil; 2 Pernambuco State Center of Hematology and Hemotherapy, Recife, Pernambuco, Brazil; 3 Department of Infectious Disease/Institute of Tropical Medicine, University of São Paulo, São Paulo, Brazil; 4 Blood Systems Research Institute, San Francisco, California, United States of America; 5 Clinical Laboratory, Department of Pathology, Hospital das Clínicas, School of Medicine, University of São Paulo, São Paulo, Brazil; Technische Universität Dresden, Medical Faculty, Germany

## Abstract

**Background:**

The findings of frequent circulation of HIV-1 subclade F1 viruses and the scarcity of BF1 recombinant viruses based on *pol* subgenomic fragment sequencing among blood donors in Pernambuco (PE), Northeast of Brazil, were reported recently. Here, we aimed to determine whether the classification of these strains (n = 26) extends to the whole genome sequences.

**Methods:**

Five overlapping amplicons spanning the HIV near full-length genomes (NFLGs) were PCR amplified from peripheral blood mononuclear cells (PBMCs) of 26 blood donors. The amplicons were molecularly bar-coded, pooled, and sequenced by Illumina paired-end protocol. The prevalence of viral variants containing drug resistant mutations (DRMs) was compared between plasma and PBMCs.

**Results:**

Of the 26 samples studied, 20 NFLGs and 4 partial fragments were *de novo* assembled into contiguous sequences and successfully subtyped. Two distinct BF1 recombinant profiles designated CRF70_BF1 and CRF71_BF1, with 4 samples in profile I and 11 in profile II were detected and thus constitute two novel recombinant forms circulating in PE. Evidence of dual infections was detected in four patients co-infected with distinct HIV-1 subtypes. According to our estimate, the new CRF71_BF1 accounts for 10% of the HIV-1 circulating strains among blood donors in PE. Discordant data between the plasma and PBMCs-virus were found in 15 of 24 donors. Six of these strains displayed major DRMs only in PBMCs and four of which had detectable DRMs changes at prevalence between 1-20% of the sequenced population.

**Conclusions:**

The high percentage of the new RF71_BF1 and other BF1 recombinants found among blood donors in Pernambuco, coupled with high rates of transmitted DRMs and dual infections confirm the need for effective surveillance to monitor the prevalence and distribution of HIV variants in a variety of settings in Brazil.

## Introduction

One of the most prominent features of HIV-1 is the remarkable accumulation of genetic diversity in its population during the course of infection. This diversity can be attributed to the high mutation rate of reverse transcriptase (3×10^−5^ substitutions per site per generation) [1], rapid viral turnover (10^8^ to 10^9^ virions per day) [2], large number of infected cells (10^7^ to 10^8^ cells) [3], and recombination events that are taking place during replication [4]. Consequently, the HIV-1 population is composed of a swarm of highly genetically related variants, i.e. a *quasispecies*, capable of rapidly adapting to various selective pressures. This diversity has been shown to have an impact not only on viral phenotypes at the level of transmission patterns, pathogenicity and immunology but also in responses to antiretroviral therapy and vaccines [5], [6]. Nine distinct genetic subtypes, (A–D, F–H, J and K) are joined in the pandemic today by more than 70 major circulating recombinant forms (CRFs) [http://www.hiv.lanl.gov/content/sequence/HIV/CRFs/CRFs.html] and numerous unique recombinant forms (URFs) have been isolated from individual patients [7]. Recombination between the URFs and CRFs and between the existing CRFs (inter-CRF recombinants) results in emergence of novel second and third generation recombinant forms which would further continue to shape the future of HIV epidemic through the generation of other variants with improved fitness to influence viral transmissibility [8], [9]. It has been reported that recombinant viruses including the URFs and CRFs may account for at least 20% of all HIV infections [10]. The existence of recombinant viruses is an evidence of simultaneous infection of multiple viruses during a single transmission event (co-infection) or from the sequential infection of viruses during multiple transmission events (superinfection).

Brazil, the most populous country in the Latin America, is home to about one third of the people living with HIV (608,230) in Central and South America (UNAIDS. 2010–2011 Report on the Brazilian response to HIV/AIDS). HIV-1 subtype B is a major genetic clade circulating in the country but the overall prevalence of non-B strains, particularly URF BF1, C and URF BC, is increasing [11], [12], [13], [14]. Data from recent studies of the viral near full-length genomes (NFLGs) have provided evidence of Brazilian CRF strains designated as CRF28_BF, CRF29_BF, CRF39_BF, CRF40_BF, CRF46_BF and CRF31_BC [12], [13], [15], [16]. Recently, Alencar et al [17] performed a molecular epidemiological survey of HIV-1 in Brazil and analyzed the partial *pol* gene of 341 samples from seropositive blood donors collected between 2007 and 2011 from 4 Brazilian blood centers of the REDS-II (Retrovirus Epidemiological Donor Study) international program. The study reported a relatively high prevalence of subclade F1 (26 [24%] of 110), and only one case of BF1 recombinant among blood donors from Recife, Pernambuco (PE). These findings contrast with those from the previous studies of HIV-1 NFLGs in Brazil [13], [18], [19], [20], [21]. We therefore undertook this study to determine whether the classification of these strains extends to the whole genome sequences. Additionally, we aimed to compare the rate of drug resistance mutations (DRMs) in plasma bulk RNA and PBMC massively parallel sequencing (MPS) data to elucidate the differences in resistance profile between both compartments. The results revealed a considerable diversity of BF1 mosaic structures and high percentage of the new RF71_BF1 and other BF1 recombinants among blood donors in PE with high rates of transmitted DRMs and dual infections.

## Materials and Methods

### Study samples

The 24 peripheral blood mononucleated cells (PBMCs) samples reported in this study were from HIV-1 seropositive blood donors in Recife, capital of Pernambuco in the northeast region of Brazil. The rationale for collection of these samples has been previously reported [17]. No evidence of direct epidemiological linkage could be established. All study subjects provided written informed consent. Ethical approval was obtained from the local ethical review committee of the HEMOPE foundation as well as the Recipient Epidemiology and Donor Evaluation Study-II collaborating centers (Blood Systems Research Institute/University of California San Francisco) and Data Coordinating Center (Westat, Inc.) in the US.

### DNA extraction and amplification of the NFLGs

The genomic DNA used for the PCR analyses was extracted using the QIAamp blood kit (Qiagen GmbH, Hilden Germany) according to the manufacturer's instructions. The NFLGs from five overlapping fragments were obtained by PCR using the Platinum *Taq* DNA Polymerase High Fidelity (5 U/µl) (Invitrogen, Life Technologies, Carlsbad, CA) and determined by a previously reported method [12], [18]. The amplified DNA fragments from the nested PCR products were separated by gel electrophoresis and purified using Freeze ‘N Squeeze DNA Gel Extraction Spin Columns (Bio-Rad, Hercules, CA, USA). Each purified amplicon was quantified using Quant-IT HS reagents (Invitrogen, Life Technologies, Carlsbad, CA), and all five amplicons from a single viral genome were pooled together at equimolar ratios.

### Whole viral genome library preparation

Sequencing libraries were prepared as described previously [22]. Briefly, one ng of each sample amplicon pool was used in a fragmentation reaction mix using the Nextera XT DNA sample prep kit according to the manufacturer's protocol (Illumina, San Diego, CA). The tagmentation and fragmentation of each pool were simultaneously performed by incubation for 5 min at 55°C followed by incubation in neutralizing tagment buffer for 5 min at room temperature. After neutralization of the fragmented DNA, a light 12-cycle PCR was performed with Illumina Ready Mix to add Illumina flowcell adaptors, indexes and common adapters for subsequent cluster generation and sequencing. Amplified DNA library was then purified using Agencourt AMPure XP beads (Beckman Coulter), which excluded very short library fragments. Following AMPure purification, the quantity of each library was normalized to ensure equally library representation in our pooled samples. Prior to cluster generation, normalized libraries were further quantified by realtime PCR (qPCR) using the SYBR fast Illumina library quantification kit (KAPA Biosystems) following the instructions of the manufacturer. The qPCR was run on the 7500 Fast Real-Time PCR System (Applied Biosystems). The thermocycling conditions consisted of an initial denaturation step at 95°C for 5 min followed by 35 cycles of 30 s at 95°C and 45 s at 60°C. The final libraries were pooled at equimolar concentration and diluted to 4 nM. To denature the indexed DNA, 5 µL of the 4 nM library were mixed with 5 µL of 0.2 N fresh NaOH and incubated for 5 min at room temperature. 990 µL of chilled Illumina HT1 buffer was added to the denatured DNA and mixed to make a 20 pM library. After this step, 360 µL of the 20 pM library was multiplexed with 6 µL of 12.5 pM denatured PhiX control to increase sequence diversity and then mixed with 234 µL of chilled HT1 buffer to make a 12 pM sequenceable library. Finally, 600 µL of the prepared library was loaded on an Illumina MiSeq clamshell style cartridge for paired end 250 bp sequencing reads.

### Data analysis

Fastq files were generated by the Illumina MiSeq reporter for downstream analysis and validated to evaluate the distribution of quality scores and to ensure that quality scores do not drastically drop over each read. To take the sequencing error rate into account, we only considered variants detected at a frequency higher than 1% and Phred quality score of >30%, i.e., a base call accuracy of 99.9%. Validated fastq files from each viral genome were *de novo* assembled into contiguous sequences and annotated with CLC Genomics Workbench version 5.5 (CLC Bio, Aarhus, Denmark) with default parameters and were additionally assembled using Velvet implemented in the Sequencher program 5.2 (Gene Code Corp., Ann Arbor, MI). The contiguous genomic sequence from each virus strain was extracted from the assembly and used for further analysis. The full designation of samples, according to WHO-proposed nomenclature, is YYBR_PEXXX, where YY stands for the year of study, BR for Brazil, PE for Penambuco, and XXX stands for sample number.

### Screening for recombination events and identification of breakpoints

The *de novo* assembled NFLGs and partial consensus sequences were aligned with reference sequences representing subtypes A–D, F–H, J and K obtained from the Los Alamos database (http://hiv-web.lanl.gov) using MAFFT version 7 [23]. Aligned sequences were manually edited and trimmed to the minimal shared length in the BioEdit Sequence Alignment Editor Program. The gap-stripped aligned sequences were screened for the presence of recombination by the bootscan methods and similarity plots implemented in the SIMPLOT program v3.5.1 [24], [25]. The following parameters were used in bootscan method: window size, 350 bp; step size, 30 bp; the F84 model of evolution (Maximum likelihood (ML)) as a model to estimate nucleotide substitution; transition\transversion ratio, 2.0; and a bootstrap of 100 trees. In addition, the significant threshold for the bootscan was set at 70%. The jumping profile Hidden Markov Model (jpHMM) [26] was also used to confirm the recombination events and to define the recombination breakpoints according to the HXB2 coordinate system. Recombinant regions of the alignment as determined by the crossover points from the jpHMM and bootscan were analyzed separately by phylogenetic analysis. In further analysis, a network reconstruction was performed for the data set with evidence of recombination using SplitsTree4 version 4.3 [27] using the Neighbor-Net method. The NeighborNet method and the GTR+I+G distance model were used to create the network.

### Phylogenetic Analysis

ML phylogenies were constructed using the GTR+I+G substitution model and a BIONJ starting tree. Heuristic tree searches under the ML optimality criterion were performed using the NNI branch-swapping algorithm. The approximate likelihood ratio test (aLRT) based on a Shimodaira-Hasegawa-like procedure was used as a statistical test to calculate branch support. The maximum composite likelihood in MEGA version 6 [28] was used to calculate the genetic distances between and within isolates. All trees were displayed using MEGA version 6.0 software.

### Genotyping Analysis

For provirus sequences generated in this study, the MPS reads of partial *pol* gene associated with DRMs in the protease and reverse transcriptase regions of the HIV-1 genome of each sample were aligned to their corresponding consensus sequence using the CLC Genomics Workbench version 5.5 (CLC Bio, Aarhus, Denmark). The minority HIV-1 resistant variants were identified using a threshold of >1.0% of the reads sequenced. Reads with <1% were discarded to account for potential errors due to the error rate of PCR. Amino acid positions including all listed major mutations and minor mutations associated with drug resistance were identified according to the IAS-USA 2011 and Stanford HIV drug resistance database. Due to the polymorphic nature of most minor protease substitutions, we only considered major mutations as evidence of transmission of drug resistance. The list of the NRTIs resistance related sites **41**, 62, **65**, **67**, **69, 70**, 71, 74, 75, 77, 115, 116, **151**, **184**, **210**, **215** and **219**; NNRTIs resistance related sites 90, 98, **100, 101, 103, 106**, 108, 138, 179, **181, 188**, **190**,221, 225, 227, **230**; PIs resistance related sites 10, 16, 11, 20, 24, **30, 32, 33**, 35, 36, 43, **46, 47, 48**, **50**, 53, **54**, **58**, 60, 62, 63, 64, 69, 71, 73, **74**, **76**, 77, **82**, 83, **84**, 85, **88**, 89, **90** and 93. Bolded numbers are major drug resistance mutation sites.

### GenBank accession numbers

All consensus genome assemblies generated in this study were submitted to NCBI's GenBank database (KJ849757 - KJ849783). The two distinct profiles of BF1 identical recombinant structure were registered with the Los Alamos National Database as CRF70_BF1 and CRF71_BF1.

## Results

A total of 26 strains preliminarily classified as subclade F1 (n = 25) and BF1 recombinant (n = 1) by sequence analysis of a partial *pol* region in a previous study [17] were corroborated by further phylogenetic analysis of the NFLGs and larger partial fragments. Of note, one BF1 sample (10BR_PE059) was erroneously classified as F1 in our previous study. This sequence was thus corrected in this study and used for further analyses. Sequences were obtained for all five overlapped fragments that cover the NFLGs of 20 participants. Partial sequences were obtained from four fragments derived from 4 samples as shown in **Table 1**. Two samples did not amplify for any fragment. This might be a result of technical difficulties in recovering the provirus, but it is also possible that cells other than PBMCs are infected during the early stages of HIV infection. For the purposes of this report, these 2 samples were not considered further, and the analyses include only the 24 samples whose subtypes were successfully determined.

**Table 1:** The near full-length genomic (NFLG) and partial fragments subtyping of HIV-from plasma and blood samples.

Sample ID	Sequence Fragment					Subtype		Number of Reads	Av. coverage
	A_(546–2598)_	B1_(2157–3791)_	B2_(3236–5220)_	C_(4890–7808)_	D_(7719–9537)_	Plasma	Provirus		
10BR_PE002	-	+	+	+	+	F	BF1	145757	21.299
10BR_PE004	+	+	+	+	+	F	BF1_CRF70	30843	2625
10BR_PE008	+	+	+	+	+	F	BF1_CRF71	111362	2564
10BR_PE009	+	+	+	+	+	F	BF1_CRF71	23923	5624
10BR_PE016	+	+	+	+	+	F	BF1_CRF70	29409	584
10BR_PE025	+	+	+	+	+	F	BF1_CRF70	84718	2173
10BR_PE026	+	+	-	+	+	F	BF1_CRF71	20503	2635
10BR_PE032	+	+	+	-	+	F	BF1	55741	6084
10BR_PE053	+	+	+	+	+	F	B	78936	3697
10BR_PE059^1^	+	+	+	+	+	F	BF1	30166	2619
10BR_PE064	+	+	+	+	+	F	BF1_CRF71	219755	5608
10BR_PE066	+	+	+	+	+	F	BF1_CRF71	32024	6701
10BR_PE071	+	+	+	+	+	F	BF1_CRF71	98156	5578
10BR_PE073	+	+	+	+	-	F	BF1	47041	1365
10BR_PE084	+	+	+	+	+	F	BF1_CRF71	15195	2034
10BR_PE085	-	-	-	-	-	F		-	-
10BR_PE086	+	+	+	+	+	F	BF1	15348	1106
10BR_PE087	-	-	-	-	-	F		-	-
10BR_PE088	+	+	+	+	+	F	BF1_CRF71	42801	700
10BR_PE090	+	+	+	+	+	F	BF1_CRF71	18052	187
10BR_PE092	+	+	+	+	+	F	BF1_CRF71	4546	201
10BR_PE094	+	+	+	+	+	F	BF1_CRF71	273899	6870
10BR_PE102	+	+	+	+	+	F	BF1	494376	7257
10BR_PE104	+	+	+	+	+	BF1	B/BF1	26901	3324
10BR_PE107	+	+	+	+	+	F	F1	78655	2159
10BR_PE109	+	+	+	+	+	F	BF1_CRF70	165634	6190

1 Variant was erroneously classified as F1 in our previous study.

**Figure 1:**
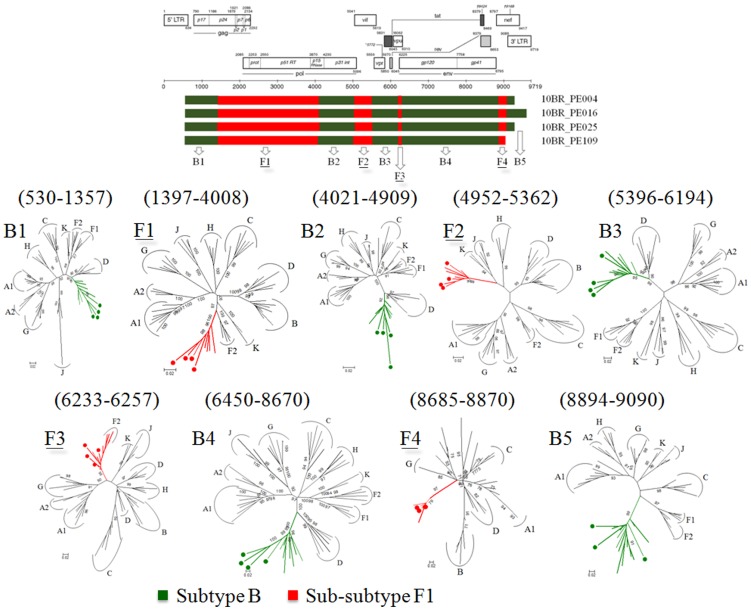
Schematic representation of NFLGs structure and breakpoint profiles with confirmatory phylogenetic trees of the four sequences identified in this study as CRF70_BF1 (colored circles). The phylogenetic trees of the nine mosaic segments defined by jpHMM, similarity plot and bootscan analysis were constructed with PHYML v.3.0. For clarity purposes, the trees were midpoint rooted. The region of subclade F1 and subtypes B are as indicated at the bottom. Positions of breakpoints are numbered relative to the HXB2 numbering system. The approximate likelihood ratio test (aLRT) values of ≥90% are indicated at nodes. The scale bar represents 0.02 nucleotide substitution per site. The results from the ML analysis were sufficiently robust to confirm the structure for the four specimens that were suggested by the recombination analysis.

**Figure 2:**
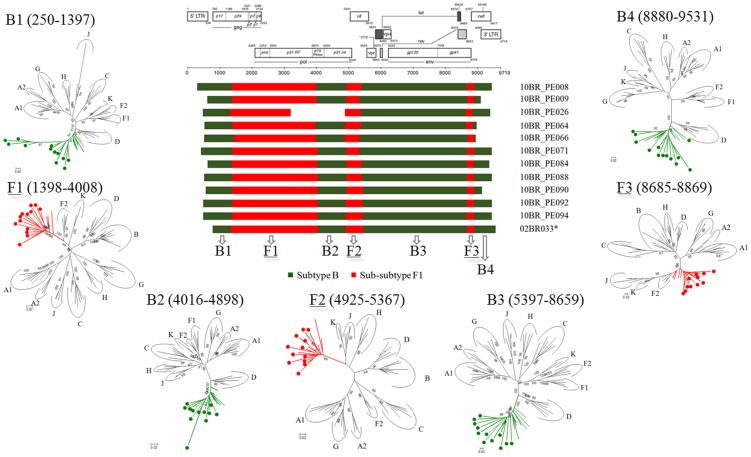
Schematic representation of NFLGs structure and breakpoint profiles with confirmatory phylogenetic trees of the twelve sequences identified in this study as CRF70_BF1 (colored circles). For patient 10BR_PE026, we were unable to amplify fragment designated as B2 and therefore we could not investigate the location of breakpoint at the 5′ of fragment B2. All ML phylogenic trees were constructed using the PHYML v.3.0 package. The region of subclade F1 and subtypes B are indicated at the bottom. Positions of breakpoints are numbered relative to the HXB2 numbering system. For clarity purposes, the trees were midpoint rooted. The approximate likelihood ratio test (aLRT) values of ≥90% are indicated at nodes. The scale bar represents 0.02 nucleotide substitution per site. The results from the ML analysis were sufficiently robust to confirm the structure for the twelve specimens that were suggested by the recombination analysis.

**Figure 3:**
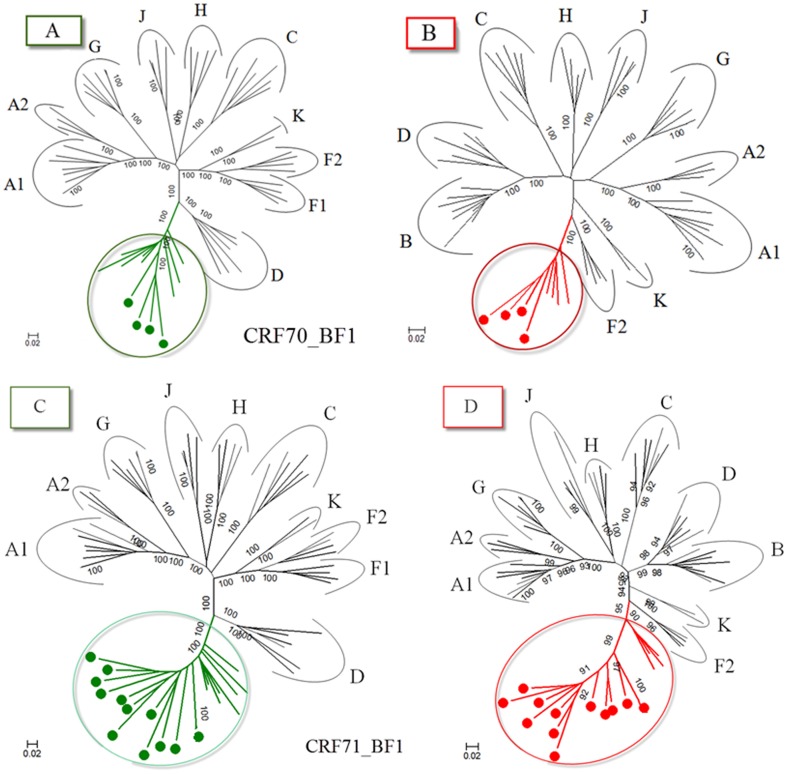
ML phylogenetic tree from concatenated regions assigned as subtype B and F1 from four CRF70_BF1 (3A and 3B) and twelve CRF71_BF1 (3C and 3D) isolates as defined by recombination analysis. Sequences from each region were aligned with reference sequences representing subtypes A–D, F–H, J and K obtained from the Los Alamos database (http://hiv-web.lanl.gov). For clarity purposes, the trees were midpoint rooted. The approximate likelihood ratio test (aLRT) values of ≥90% are indicated at nodes. The scale bar represents 0.02 nucleotide substitution per site. The results from this analysis revealed that each segment of the CRF70_BF1 (3A and 3B) and twelve CRF71_BF1 (3C and 3D) viruses was found to cluster with corresponding segments of subtype B or F1 viruses in agreement with the subtype assigned by recombination analysis.

**Figure 4:**
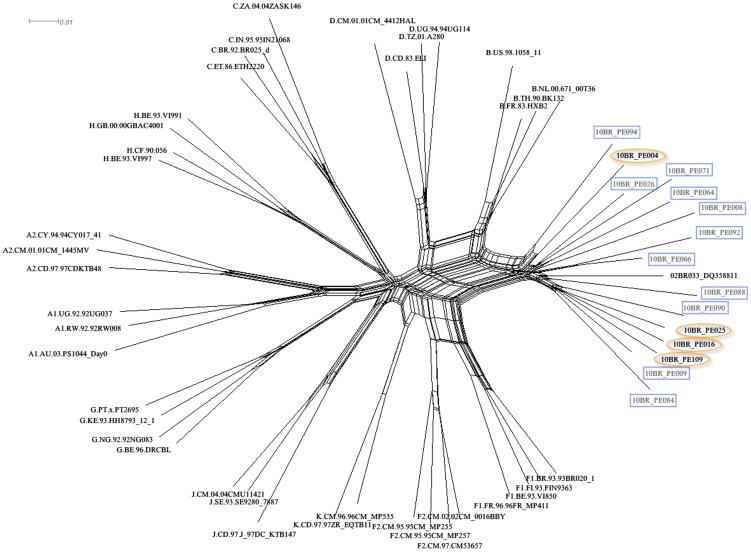
Split networks for four sequences of CRF_70BF1 (marked with orange circles) and eleven CRF_71BF1 (marked with blue boxes) and representatives of HIV subtypes A–K sequences from the Los Alamos database (http://hiv-web.lanl.gov). The splits graphs used distances computed under the GTR+I+G model. The scale bar represents 0.01 nucleotide substitution per site. The results demonstrate that sequences within each of CRF did not group directly with each other, but they were present as one group of sequences within a cluster between subtype B and subclade F1.

Analysis of the proviral NFLGs and partial consensus sequences revealed all isolates retain intact reading frames for a majority of their genes and no gross deletions or rearrangements were observed. Recombination analysis from each strain shows two distinct recombinant profiles with 4 samples in profile I and 11 in profile II. The recombinant genomes of both profiles essentially consisted of subclades F1 and subtype B as parental sequences and appeared different from all previously documented CRFs in Brazil and South America. Plausible breakpoints identified in each profile using bootscanning analyses were consistent with those identified using jpHMM (schematically illustrated in Figure 1 and 2). Therefore, the new recombinants strains in profile I and II are now designated CRF70_BF1 and CRF71_BF1, respectively. All of the breakpoints in the two CRFs were mapped and compared. The results revealed great similarity in structure between the two CRFs. Among the sub-subtype F1 stretches, identified by letter F1 through F4 and F1 through F3 in CRF70_BF1 and CRF71_BF1, respectively (Figure 1 and 2), fragments F1, F2 and F4 appear to be in the same locations. For patient 10BR_PE026, we were unable to amplify fragment designated as B2 in **Table 1** (position 3236–5220) and therefore could not investigate the location of breakpoint at the 5′ of fragment B2 as depicted in Figure 2. Different from CRF71_BF1, CRF70_BF1 appears to contain one too short sub-subtype F1 fragment within the *Vpu* gene (34 bp, nucleotide 6233–6266 according to position in HXB2 GenBank accession no. K03455) interspersed with subtype B. In attempt to find sequences with recombinant structure similar to any of the strains in the two CRFs, multiple sequence alignments including all published BF1 unique NFLGs sequences were built, aligned and subjected to bootscan and automated jpHMM. The results showed that the CRF71_BF1 sequences had a mosaic sequence pattern nearly identical to the previously published Brazilian BF1 isolate 02BR033 (GenBank: DQ358811) in sample collected in 2002 from patient living in São Paulo, Southeast of Brazil [13]. To further test for recombination, ML phylogenetic trees were inferred for the regions of nucleotide sequence on either side of the breakpoints detected by bootscan and jpHMM methods. As shown in Figure 1 and 2, the exploratory tree analysis revealed that each fragment of recombinant virus from each CRF clustered tightly (>90% aLRT) with corresponding fragments of subtype B or F1 reference sequences in agreement with the subtype assigned by recombination analysis. The only disagreement between the recombination and exploratory tree analyses was for a short CRF70_BF1 fragment in the middle of the *Vpu* region (F3 in Figure 2). Despite the fact that F3 fragment in CRF70_BF1 strains have shorter sequences and some group M variants cannot resolve some of the internal nodes, all of the CRF70_BF1 can resolve the terminal nodes and appeared more closely related phylogentically to subclade F1 than the other groups of reference sequences. To increase the phylogenetic signal, ML trees were performed on concatenated data of discontinuous subtype B and F1 fragment from CRF70_BF1 (Figure 3A and 3B) and CRF71_BF1 (Figure 3A and 3B), which was well confirmed by aLRT values above 90% as shown in Figure 3. Split decomposition was then used to visualize the relationship of the two CRFs (Figure 4). While sequences within each of these two CRF did not group directly with each other, they were present as one group of sequences within a cluster between subtype B and F1. The observed mean intrasubtype genetic distances were 8.2% (range 6.7–10.0%) and 8.7% (range 6.2–9.9%) for CRF70_BF1 and CRF71_BF1 strains, respectively, and closer to the intersubtype distance between both CRFs (8.3%).

**Figure 5:**
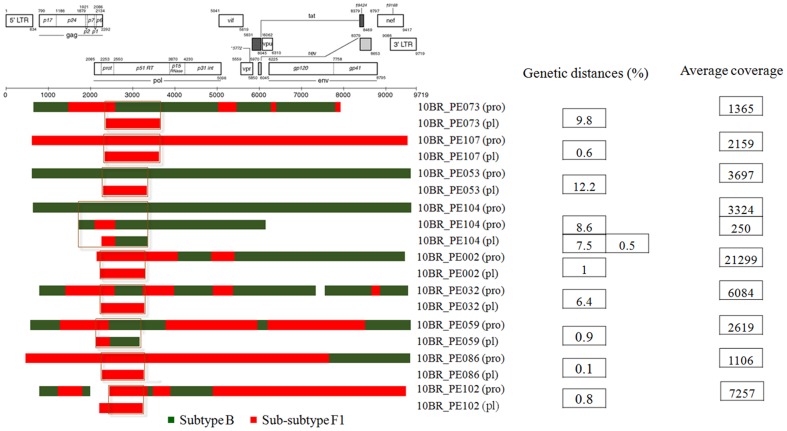
Schematic representation of the NFLGs, partial structure and breakpoint profiles of the BF1 sequences identified in this study from proviral DNA generated by deep sequencing approach and previously published cell free viruses generated by bulk sequencing approach. Sequences were mapped relative to the HXB2 numbering system. Genetic distances of overlapping regions (marked with orange boxes) between sequences from plasma and PBMCs together with the overall mean coverage depth are demonstrated. Distances were computed using the maximum composite likelihood method in MEGA version 6 [28]. As depicted in the figure, the intra-individual plasma and proviral sequence variation in four patients (10BR_PE073, 10BR_PE053, 10BR_PE104 and 10BR_PE032) in the partial *pol* regions (marked with orange boxes) were remarkably high. These results may indicate that the plasma viruses were derived from a population significantly distinct from those of the cellular sources; a result consistent with dual infection with different subtype. In sample 10BR_PE104, MPS data revealed the existence of subtype B NFLGs and a second BF1 recombinant strain (4450 bp) almost identical to the plasma virus in the same region.

**Figure 6:**
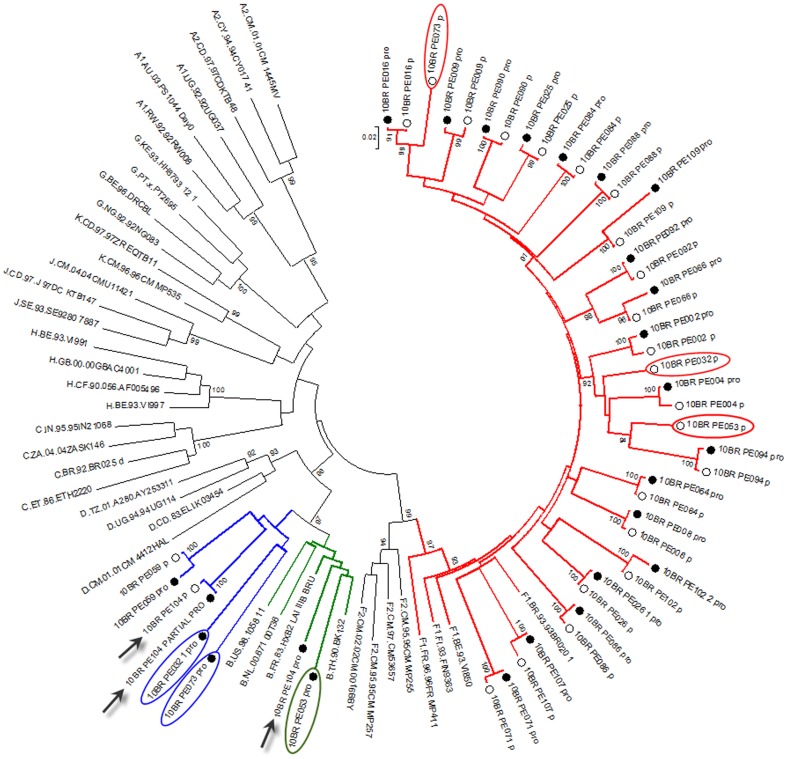
Comparison of phylogenetic clustering profile of sequences from both plasma (empty circles) and provirus (black circles) and other HIV-1 reference sequences from the Los Alamos HIV-1 database representing 11 genetic subtypes. Viral sequences from both compartments were aligned with the complete set of reference sequences obtained from the Los Alamos database (http://hiv-web.lanl.gov). Green, blue and red colored branches represent subtype B, BF1 recombinants, and subclade F1, respectively. Sequence with discordant results between PBMCs and plasma are marked with blue and red oval circles, respectively. For purposes of clarity, the tree was midpoint rooted. The approximate likelihood ratio test (aLRT) values of ≥90% are indicated at nodes. The scale bar represents 0.02 nucleotide substitutions per site. Except subjects 10BR_PE073, 10BR_PE053, 10BR_PE104 and 10BR_PE032, each patient forms a tight cluster and is distinct from other subjects with aLRT SH-like supports >95% for all inter-subject clusters. The results from the ML analysis added further support to the results depicted in figure and were sufficiently robust to confirm the event of dual infection with different subtype. In case 10BR_PE104 (indicated by black arrow), MPS data revealed the existence of subtype B NFLGs and a second BF1 recombinant strain almost identical to the plasma virus in the same region.

Beside the CRFs, in five of the seven strains initially classified as infected with subclade F1, subtype B fragments were detected and appeared to represent URFs strains (Figure 5). The relationships of the viral sequences from patients' PBMCs to the sequences obtained from the corresponding RNA viruses within the same regions were examined for each patient to roughly assess the viral diversity in both compartments (Figure 6). Surprisingly, the intra-individual plasma and proviral sequence variation in four patients (10BR_PE073, 10BR_PE053, 10BR_PE104 and 10BR_PE032) in the partial *pol* regions depicted in Figure 5 (marked with orange boxes) were remarkably high, indicating that the plasma viruses were derived from a population significantly distinct from those of the cellular sources; a result consistent with dual infection with different subtypes (Figure 5 and 6). Except those with dual infections, all the other sequences from both compartments were located close to one another on the same branch and had plasma RNA and proviral DNA variation only ranging between 0.1–0.8% (Figure 4). In the case of subject 10BR_PE104, MPS data revealed a mixture of two distinct consensus sequences, one NFLGs from strain B and a second recombinant of strains B and F1 (4450 bp) almost identical to the plasma virus in the same region. The BF1 segment was present in 7% of MPS reads with a median coverage of 250 reads in the sample from subject 10BR_PE104, while the NFLGs present in 93% of MPS reads with a median coverage of 7250 reads. Thus, the low BF1 copies would not have been detectable using the provirus traditional bulk sequencing approaches. Again, these data confirms that infection in this subject was founded by two genetic lineages. In Figure 5, there were three samples (10BR_PE002 (pro), 10BR_PE032 [17], and 10BR_PE102 [17], which shared one breakpoint at the integrase gene (position 2393–2462 nt), similar to the third breakpoint of the CRF71_BF variants (5′ of fragment F2, Figure 2). This increased prevalence of genetic breakpoints in the integrase region may indicate a possible preference region for recombination to occur.

Three viruses that were classified as BF1 (n = 2; 10BR_PE059, 10BR_PE086) and F1 (10BR_PE107) on partial *pol* analysis were confirmed by NFLGs analysis (Figure 5).

As stated before, the 26 samples selected to this study are derived from 110 blood donors from Recife PE, and all were identified as infected with subclade F1 except two donors who were found to be infected with BF1 recombinant viruses. Genotyping based on NFLGs or long partial fragments of these samples was successful in 24 donors, of whom 11 were infected with CRF71_BF1, 4 with CRF70_BF1, 6 BF1 URF, 1 subtype B, 1 subtype F1, and 1 dually infected with BF1 and B viruses. Based on this analysis, it is evident that non-recombinant subclade F1 accounts for <1% of HIV-1 subtype's circulating in PE and that CRF71_BF1 is responsible for 50% (11/22) of infections caused by BF1 recombinants.

We also aimed in this study to analyze the emergence of proviral HIV DRMs detected by MPS and compare the results with the plasma HIV DRMs detected by previously described plasma bulk sequencing of the same blood donors group to better understand the resistance profile between the two compartments. A summary of these results are provided in **Table 2**. Discordant data between the cell free viruses and the PBMC-viruses were found in 15 participants. Six of these strains displayed major amino-acid changes only in the cellular compartment and four of which had detectable major amino-acid changes at prevalence between 1–20% of the sequenced population. Neither major amino-acid changes in the protease nor in the reverse transcriptase coding region were detectable by plasma bulk sequencing. Eleven and two DRMs in the protease and reverse transcriptase regions, respectively, were detected in plasma bulk sequencing but were not observed in MPS data. For the coding region of the protease gene, major PI resistance mutations, namely, M46I, and L33F were detected in four blood donors. As shown in **Table 2**, the overall frequency of minor mutations in both compartments in the protease gene was significantly greater than that detected in reverse transcriptase. Although these mutations were mostly polymorphisms and not directly responsible for drug resistance but they are able to compensate the low protease processivity caused by primary mutations [29]. The proviral DNA MPS analysis in the reverse transcriptase region showed major mutations at the following codons in five patients: M184I, M230I, and E138K. It is well known that HIV-1 harboring the M184I/V mutations has a low viral fitness because of deficient dNTP usage and that the E138K mutation can compensate for the deficit in dNTP usage associated with the M184I/V mutations [30]. The compensatory substitution at codon 230 in motif E contributes to reduced viral replication and has also been shown to confer resistance to all currently available NNRTIs in both phenotypic and biochemical assays [31].

**Table 2:** Drug resistance mutations detected with bulk sequencing (plasma) and deep sequencing (PBMCs).

Sample ID	Protease gene coding region		Reverse transcriptase gene coding region(nRTIs)		Reverse transcriptase gene coding region (NNRTIs)	
	PLASMA (Bulk sequencing)	PBMC (Deep sequencing)	PLASMA (Bulk secuencing)	PBMC (Deep sequencing)	PLASMA (Bulk secuencing)	PBMC (Deep sequencing)
10BR_PE002^•^	M36I, V77I^¶^, L89M	M36I,*V82I*, L89M		M184I*		M230I*
10BR_PE004^•^	M36I, V77I, L89M	M36I,***M46I***,V77I, L89M			V106I^¶^	
10BR_PE008^•^	M36I, D60E, L89M	M36I, D60E, L89M				*V106I*
10BR_PE009^•^	L10V, M36I, L89M	L10V, M36I, L89M		F77L* 1, M184I*		M230I*
10BR_PE016^•^	L10V, M36I, L89M	L10V, *G16E*, M36I, L89M				
10BR_PE025	L10V, K20R, M36I, L89M	L10V, K20R, M36I, L89M				
10BR_PE026	M36L, L89M	M36I, L89M				
10BR_PE032^•^	L10V, M36I, L89M	L10V, L33F*, M36I, L89M		M184I*		V108I, M230I*
10BR_PE053^•^	M36I^¶^, L89M^¶^	*V77I*				
10BR_PE059	L10I, M36I, L89M	L10I, M36I, L89M				
10BR_PE064	M36I, L89M	M36I, L89M				
10BR_PE066^•^	L10V, K20R^¶^, M36I, D60E^¶^, I62V, L63P, L89M, I93L	L10V, M36I, D60E, I62V, L89M, I93L				
10BR_PE071^•^	M36I	M36I		F77L* 1		
10BR_PE073	M36I, I64M	M36I, I64M				
10BR_PE084	L10V, M36I, I64M, L89M	L10V, M36I, I64M, L89M				
10BR_PE086^•^	L10I^¶^, K20R, M36I, I62V, L89M, I93L	K20R, M36I, I62V, L89M, I93L				
10BR_PE088	M36I, V82I, L89M	M36I, V82I, L89M			V106I	V106I
10BR_PE090^•^	L10I, M36I, L89M	L10I,M36I, M46I*, L89M		M184I*		
10BR_PE092	M36I, D60E, I62V, I64M, L89M	M36I, D60E, I62V, I64M, L89M				
10BR_PE094	K20R, M36I, L89M	K20R, M36I, L89M				
10BR_PE102^•^	L10I^¶^, M36I^¶^, I64L^¶^					
10BR_PE104^•^	K20R^¶^, M36I	M36I, *L63P*, *V77I, I93L*				
10BR_PE107^•^	M36I, I62V, L63P^¶^, L89M	M36I, I62V, L89M			V108I^¶^	
10BR_PE109^•^	K20M, M36I, D60E, L63P, L89M	K20M, M36I, M46I*, D60E, L63P, L89M		F77L* 1		*E138K*

Samples displayed discordant genotypic data between the cell free viruses and the PBMC-viruses are marked by black dots, Major mutations are marked in boldface type, Major drug resistance mutations at prevalence >20% of the sequenced population are underlined, Major drug resistance mutations at prevalence <20% of the sequenced population are marked by star symbol, Mutations detect only in PBMCs are marked in italic face type.

1 Transmitted drug resistance mutation, Mutations detect only in plasma are marked by pilcrow symbol.

## Discussion

This study describes the MPS of proviral NFLGs and larger fragment from 26 well sampled groups of blood donors from PE who had previously been diagnosed as infected with subclade F1 (n = 25) and BF1 recombinant (n = 1) based on *pol* subgenomic fragment from cell free viruses using conventional bulk sequencing. The most remarkable observations in this study are that 23 of the 24 donors in whom genotyping was successful infected with HIV-1 BF1 recombinant variants. Of these, two novel BF1 CRFs with high genetic diversities that exceed >8% difference at both inter- and intra-host level were identified. These results suggest that both CRFs have been in circulation early in the epidemic and have been evolving independently ever since. Based on the similarity of their recombination profile, it is tempting to speculate that the CRF70_BF1 variants were old “second-generation” recombinants of CRF71_BF1 circulating in PE. Our estimates indicated that the CRF71_BF1 variants are responsible of 50% of infection caused by BF1 recombinants among blood donors in this region of northeast of Brazil. Moreover, if we assume no recombination in the remainder of the genome characterized as subtype B in the previous study, then the prevalence of BF1 recombinant variants is estimated at 20.4% (22/108) and CRF71_BF1 at 10.2% (11/108) of HIV-1 strains circulating among blood donors in PE. Given the high prevalence of CRF71_BF1 observed among the low risk blood donors, it may be suspected that a high prevalence for this variant could be found among other high-risk groups with tight transmission chain and that it has been able to break the transmission barrier from high-risk groups into the general population. Moreover, detection of both CRFs indicates that these variants are actively competing with other BF1 recombinants and other HIV-1 subtypes circulating in this region.

The proportion of BF1 variants described in this study is much greater than the rate reported in 169 individuals recently diagnosed as seropositive for HIV1 (2.8%) in the Metropolitan Region of Recife, Northeastern of Brazil [32] and in other study of 84 patients chronically infected with HIV (3.6%) naïve to antiretroviral treatment [33]. This difference is not surprising, because small fragments from different regions of HIV genomes were characterized in the previous studies while we used larger overlapped fragments to sequence the NFLGs, which is the preferred method for accurate characterization of HIV-1 isolates. Thus, the previous studies are likely to have missed the recombinants samples. Despite the high rate of recombination as estimated using our fairly conservative assumptions, it is probable that our results have underestimated the true rate of infection with BF1 recombinant viruses; particularly our study was limited to donors infected with F1 and BF1 with partially sequenced viral fragments. Thus, it is possible that the BF1 infection in this group will be higher than what was observed if we had sequenced the virus NFLGs in all the 80 subtype B infected donors described in the previous study.

Additional observations of this study are the description of mixed infections with B, F1 and/or BF1recombinants. The use of MPS technology enhanced our power to determine the dual infection of larger fragment of BF1 and genuine NFLGs of subtype B in the PBMCs of patient 10BR_PE104. The BF1 recombination profile was almost identical to the plasma virus in the same region demonstrating an intra-individual plasma and proviral sequence variation of only 0.5%. Thus, it is possible to assume that the primary infected PBMCs harboring the BF1 recombinants in this patient were likely the source of the plasma circulating viral sequences. The observation of an intact NFLGs of subtype B in the PBMCs of patient 10BR_PE104 may argue against the inactivity of this provirus but may agree with assumption that subtype B provirus may be integrated into the chromosome at a site at which its expression is prevented, or it may be transcriptionally inactive by virtue of being extrachromosomal [34]. It is also possible that the adaptation of the B strains to the PBMC in this patient allows them to avoid competitive exclusion by the dominant strain in plasma. In the other three samples with dual infections, subtype F1 seen in the plasma were completely absent in the PBMCs. Discordances in the HIV subtypes in both compartments may suggest differential sources of infecting viruses. It is also conceivable that the discordances in the HIV subtypes in PBMCs and plasma are likely due to low-level minority strains present as B/BF1 variants that are not detected with bulk plasma sequencing or that the F1 viruses shed in the plasma were more fit. The evidence of dual infections in this study adds support to previous studies [35], [36], [37], [38], [39], as this event is far more common in Brazil where these subtypes co-circulate. Furthermore, these data agree with the consensus that the presence of two or more HIV-1 subtypes within an infected individual is relatively frequent [40], [41]. It is unclear from this study whether the occurrence of HIV multiple distinct strains was the result of superinfection with a second variant at a later time, or whether simultaneous infection with multiple viral strains occurred during a single transmission event. However, the circulation of multiple subtypes in Brazil fortifies the possibility of both scenarios. The overall results indicate that the rate of HIV-1 mixed infections within this Brazilian group is higher than 16%. From these results, we believed that the use of PBMC DNA in addition to plasma RNA is expected to provide highest sensitivity to detect mixed infection via population MPS. Whether dual infection and/or recombination had an impact on the clinical outcomes of the blood donors in this study was unknown, since the available clinical data resulted from one assessment that seek to understand risk exposures and motivations to donate blood.

Other important observation of this study is the underestimation of transmitted resistance obtained by routine plasma analysis that is revealed by the examination of the MPS populations of the archived proviruses in PBMCs. In this study, comparing both sources would have detected 11 DNA provirus disclosed DRMs by MPS not detected by routine plasma bulk analysis. In this study, eleven DNA provirus sequence had detectable DRMs previously missed by plasma bulk analysis. Six of these strains had 17 DRMs only in the PBMCs and four of which had detectable major DRMs at prevalence between 1–20% of the MPS population. These results support the previous observations that standard bulk sequencing cannot fully access the spectrum of viral variants archived in the proviral DNA [42]. The relatively higher proportion of recently infected donors carried low-abundance resistance mutations leads us to believe that the rate of the current transmitted DRMs is underestimated. It is well known that conventional Sanger sequencing of bulk PCR products are limited to the detection of high-frequency variants that present in greater than 20% of the sequenced viral population [43], [44]. However, we were able to detect additional DRMs at prevalence higher than 20% of the MPS provirus variants that had not been identified using plasma bulk sequencing. This result probably indicates that MPS approach permit characterization of considerable heterogeneity in the diversity and frequency variations in the proviral DNA [45]. Since the time of infection is unknown, it possible that some of the transmitted mutants in cell free viruses may disappear over time while persisting in cellular HIV-1 DNA [46], [47], [48]. All together, these results justify the inclusion of proviral DNA from PMBCs as a valuable source for resistance analysis, which is in agreement with previous reports [49], [50]. On the other hand, we expected that our MPS approach would detect the entire mutations spectrum from the amplified and sequenced viral population that was displayed by conventional sequencing of the cell free viruses, but this does not hold true for some mutations. Seven patient samples with some detectable DRMs by bulk sequencing approach were not observed in PBMCs reads obtained through the MPS approach. One possible explanation beside the limiting factors intrinsic to PCR is that our MPS approach of the amplified fragments is not sufficiently sensitive to detect all DRMs. It is also possible that the discordant genotypes in these patients are due to dual infection. This was confirmed in patients 10BR_PE053 and 10BR_PE104 who displayed two phylogenetically distinct populations in the sequenced regions.

Apart from small sample size, the present study was limited by the investigation of only the HIV-1 proviruses. Although plasma HIV-1 RNA remains the material of choice for the determination of drug-resistant mutations and guiding therapeutic decisions [51], [52] the proviral PBMC DNA sequence can contain a variety of multiple DRMs that are not present in plasma [47], [53]. This, combined with the stability of DNA compared with RNA [54], and the fact that HIV DNA recovered from the proviral compartment can reliably be used for the determination of DRMs in treatment naïve patients [55] influenced our decision to use proviral DNA in this study.

Finally, needless to say, HIV phylogenetic analysis based on the complete genome sequences is more reliable than that of the *pol* or other small partial fragment alone. The high percentage of the new CRF71_BF1 and other BF1 recombinants found among blood donors in Pernambuco, coupled with high rates of transmitted DRMs and dual infections confirm the need for effective surveillance to monitor the prevalence and distribution of HIV variants in a variety of settings in Brazil.
